# The Book World of Medicine and Science

**Published:** 1907-07-27

**Authors:** 


### The Book World of Medicine and Science.

### THE OXFORD MEDICAL PUBLICATIONS.—SOME ADDITIONAL VOLUMES.

Auscultation and Percussion. By Samuel Gee, M.D. (London : Henry Frowde, Oxford University Press, and Hodder and Stoughton, Warwick Square, E.C. 5s. net.)

Heart Disease and Thoracic Aneurysm. By F. J. Poynton, M.D. (London : Henry Frowde, Oxford University Press, and Hodder and Stoughton, Warwick Square, E.C. 5s. net.)

Clinical Lectures and Addresses on Surgery. By C. B. Lockwood, F.R.C.S. (London : Henry Frowde, University Press, and Hodder and Stoughton, Warwick Square, E.C. 5s. net.)

These three little volumes belong to the series of "Oxford Medical Publications," and each of them appears to us to fulfil the conditions laid down by the editors of the series in their attempt to secure uniformity of treatment and to maintain a high standard of practical excellence.

Dr. Gee's "Auscultation and Percussion," of which this volume is the fifth edition, is too well known, both to students and practitioners, to need much introduction here. It is in every way an eminently practical book, and if it lacks at times the charm and felicity of phrase that distinguished the author's "Aphorisms," it shares with the latter the clearness and lucidity which are characteristics of Dr. Gee's writings. Not the least valuable part of it is the list of authorities, appended to each page almost, and giving the reader a bibliography which, although condensed, is sufficiently accurate and catholic to satisfy any further craving for historical facts. This little book will continue to be one of the best short treatises on the art of clinical examination by those methods with which it deals.

In "Heart Disease and Thoracic Aneurysm" Dr. Poynton attempts the impossible feat of giving the student or practitioner details of the protean aspects of cardiac disease within the limits of 300 odd pages. So far as it was possible to achieve success in this endeavour, Dr. Poynton has succeeded, but his book compares unfavourably, in some respects at least, with the competitors it will have to meet in the field. It it clearly written, in a pleasant though somewhat cramped style, and it is particularly full and praiseworthily clear on the oft-neglected subject of treatment. Indeed, the treatment of many of the conditions described is given in greater detail, and with more attention to debatable points than in larger and more ambitious text-books. The description of each condition is conscientiously done, and a close perusal of the book fails to reveal more than a few slight points on which a difference of opinion might exist between author and reader. An appendix gives not only some useful formulæ and prescriptions useful in the treatment of cardiac cases, but explains the other methods of treatment, such as the Schott and the Nauheim. The book is copiously illustrated, the photographs of pathological interest being especially good.

Mr. Lockwood's clinical lectures, which are reprints in volume form of a series that has already seen the light in the "Clinical Journal," are eminently worth the reading. We have found the books of the Oxford Medical Manuals, generally considered exceptionally good, and it is therefore no mean praise to state that "Clinical Lectures and Addresses on Surgery" stands among the first of these volumes in point of excellence. A redundancy here and there, such as will necessarily occur in lectures delivered to a class where repetition, and repetition only, can fix the point in the hearer's mind, impairs the smoothness of the style at times; but the subject-matter is so interesting, and Mr. Lockwood's remarks are so instructive, that one gladly pardons these trifling slips in the editing. Every practitioner should read and ponder over the two introductory chapters, especially to that devoted to "Clinical Reasoning"; and there is no chapter from which he will not be able to glean a point of practical value, worth underlining or transfer to a notebook. For examination purposes Mr. Lockwood's book will not find favour with the student; to the practitioner, however, it will be welcome, not only because it deals with subjects not usually considered at such length as their importance warrants, but equally much because it draws upon a large and varied surgical experience.

The three volumes we have so briefly reviewed are excellent additions to a very excellent series, and as such are eminently suitable for the general practitioner's library.

---

Pulmonary Phthisis : Its Diagnosis, Prognosis, and Treatment. By H. Hyslop Thomson, M.D. (London : John Bale, Sons and Danielsson, 83 Great Titchfield Street, W. 5s. net.)

There are already several good manuals on the subject with which this volume deals, and in reviewing Dr. Thomson's book the critic is tempted to draw comparison and contrast which, on the whole, would be unjustifiable. For Dr. Thomson's little brochure is not in the same rank with the larger treatises on consumption. It is an epitome of pulmonary tuberculosis—we confess to a dislike of the term "Pulmonary Phthisis"—of its diagnosis, prognosis, and treatment by one who has had a large and varied experience of sanatorium treatment of the disease, and whose primary object has been to deal with the disease from a practical standpoint. On the diagnosis there is little in this book which the general practitioner will find new. The *x*-rays as a help in the diagnosis of incipient lung lesions needs too expert knowledge of radiography to appeal to the average practitioner, and the author, we think, does well to dismiss it so briefly as he does. Much more important, and from the general practitioner's point of view of far greater practical value, is the chapter on Prognosis, which is very carefully written. The only omission we notice is an account of the diazo-reaction in the urine, which, however debatable its prognostic value may be, is certainly a test that can be very easily applied in general practice and from which the medical attendant may learn a good deal. On a few points Dr. Thomson might have been more explicit—as, for instance, on the pulse-rate, to which barely a page is devoted. The chapters on treatment, diet, and after-care are excellent, and the appendix contains much interesting information. The little book is thoroughly practical, well written, and informative, and will undoubtedly prove of service to the practitioner who has to deal with cases of incipient or advanced phthisis.

